# Arterio–Ureteral Fistula as a Long-term Complication Following Retroperitoneal Presacral Ganglioneuroma Resection: Case Report in an Adolescent and Review of the Literature

**DOI:** 10.1055/a-2496-5087

**Published:** 2024-12-24

**Authors:** Theresa S. Haecker, Thomas F. Krebs, Regula von Allmen, Frank-Martin Haecker

**Affiliations:** 1 Department of Pediatric Surgery, Children's Hospital of Eastern Switzerland, St. Gallen, Switzerland; 2 Department of General, Visceral, Thoracic, Transplant and Pediatric Surgery, University Hospital of Schleswig-Holstein Kiel Campus, Kiel, Germany; 3 Department of Vascular Surgery, Kantonsspital St. Gallen, St. Gallen, Switzerland; 4 Faculty of Medicine, University of Basel, Basel, Switzerland

**Keywords:** arterio–ureteral fistula, hematuria, chronic indwelling ureteral stent, pelvic surgery, adolescents

## Abstract

Arterio–ureteral fistula (AUF) is a rare condition affecting nearby adult-only patients. Patients usually present with hematuria, often starting as intermittent hematuria which frequently increases, and may lead to hemorrhagic shock. Without rapid diagnosis and prompt treatment, AUF can be lethal. Risk factors for developing an AUF include a history of pelvic surgery mainly due to cancer, a history of pelvic radiation, a history of vascular surgery, or chronic indwelling ureteral stents (CIUS). Imaging to confirm diagnosis includes angiography, computed tomography (CT) scan, or retrograde pyelography, although AUF may be missed. Therefore, even if imaging is negative, the presence of hematuria of unexplained origin in combination with mentioned risk factors is highly suspicious for AUF and must be excluded.

We report the case of a 16-year-old male patient who presented with a new onset of intermittent hematuria to our emergency room. The patient's history included previous pelvic surgery for resection of ganglioneuroma 6 years ago with bilateral replacement of the iliac artery and postoperative acute kidney failure with reconstruction of both ureters and CIUS. After the initial uneventful postoperative follow-up over 5 years, another Double J (DJ) catheter had to be placed into the right ureter due to hydronephrosis. Six weeks later, the patient presented with intermittent hematuria. Despite negative imaging, we performed immediate surgical exploration confirming the diagnosis of AUF. To the best of our knowledge, this is the first case of AUF under the age of 18 years reported in the literature.

In conclusion, in patients with macrohematuria and a history of the abovementioned risk factors, AUF has to be kept in mind and must be reliably excluded.

## Importance for a Pediatric Surgeon

Arterio–ureteral fistula (AUF) represents a very rare condition. The condition may lead to hemorrhagic shock, and without rapid diagnosis and prompt treatment, it can be lethal. Possible risk factors for developing an AUF include a medical history of pelvic surgery, a history of pelvic radiation, and a history of vascular surgery, which is very uncommon in the field of pediatric and adolescent surgery.

## Introduction

Arterio–ureteral fistula (AUF) represents a rare condition including a pathological connection between the ureter and artery, most commonly referred to as the iliac artery or previously implanted graft. Only a few cases are reported in the literature, affecting adult-only patients. Hematuria is the leading and often only clinical symptom and can vary in intensity from intermittent to massive bleeding. Depending on the severity of the bleeding, it may be a life-threatening event. 1 2 3 4 5 6 AUF is rare, and its symptoms are unspecific. It is an uncommon and challenging clinical scenario involving the skills and experience of a urologist, a vascular surgeon, and an interventional radiologist, and it is often difficult to make the diagnosis. Furthermore, there is no standardized reliable imaging method available. Pyelography, ureterorenoscopy, and flexible cystoscopy can easily miss the diagnosis, while provocative angiography can reach a 100% sensitivity rate but may expose the patient to the risk of uncontrollable bleeding. In patients with suspected AUF, on average more than two imaging tests per patient are performed, of which only 46% may confirm the AUF. 3 Possible risk factors for developing an AUF include a medical history of pelvic surgery (HPS), history of pelvic radiation (HPR), history of vascular surgery (HVS) in the same area, and chronic indwelling ureteral stents (CIUS). These risk factors are usually noticed in adult-only patients and not adolescents. A high mortality rate is reported. 6

Reviewing the literature, we may find several AUF case reports and small case series, however due to the abovementioned reasons on patients older than 18 years of age. The incidence of pediatric and adolescent patients is unknown. We report the case of a 16-year-old male patient with HPS, HPV, and repeated CIUS, who presented with a new onset of hematuria.

## Case Report

A 16-year-old boy with a complex medical history presented to our emergency room due to a new onset of macrohematuria and pain around the bladder and right kidney. Six years before, the patient had been diagnosed with a retroperitoneal presacral ganglioneuroma, with infiltrating growth of surrounding vessels. R1 tumor resection was achieved. However, a lesion of the right common iliac artery (CIA) had to be noticed, which required replacement with a self-made bovine pericardial graft. Postoperatively, the patient developed acute kidney failure due to bilateral ureteral compression with consecutive bilateral hydronephrosis. After temporary bilateral urinary diversion with a percutaneous pigtail catheter, the right ureter was reconstructed with segment resection and end-to-end anastomosis, followed by reconstruction of the left ureter 5 weeks later. Bilateral DJ catheter placement enabled safe internal urinary drainage for 5 to 7 months. After the scheduled removal of the stents, further follow-up examinations including imaging were uneventful.

**Fig. 1:**
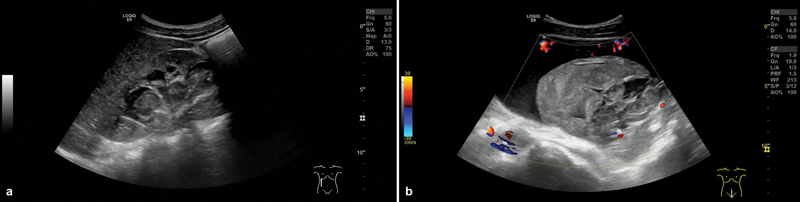
Ultrasound during the initial assessment for further clarification of the hematuria. ( **a** ) Right kidney: dilatation of the pyelon, the central calices, and the proximal ureter. Subtotal obliteration of the right renal pelvic calyx system by blood clots. DJ-catheter-tip in situ. ( **b** ) A voluminous blood clot in the dilated bladder. DJ, Double J.

Five years after primary surgery, routine follow-up sonography revealed an increasing dilatation of the right renal pelvic caliceal system. Mercaptoacetyltriglycin (MAG3)-scintigraphy confirmed impairment of the renal split function and obstruction of the enlarged right kidney. DJ-catheter placement using a resonance metallic ureteral stent (Charrière 6, 26 cm) was performed. After a short postoperative period with dysuria and flank pain managed by iv antibiotics, the patient was discharged in good condition. Six weeks later, the patient presented with intermittent macrohematuria to our emergency room. Ultrasound showed voluminous blood clots with subtotal obliteration of the right renal pelvic caliceal system ( Fig. 1a ) as well as in the enlarged urinary bladder ( Fig. 1b ), with the correct position of the DJ catheter. Transurethral urinary bladder catheter placement led to immediate improvement of the symptoms. Nevertheless, the patient was admitted for observation. A clear drop in hemoglobin 2 days later led to the indication for immediate relaparotomy. After the removal of the right-sided DJ catheter and evacuation of a large hematoma from the bladder, persistent bleeding from the ureter was noticed. The ureter was opened at the iliac crossing, and we identified an AUF with an opening at the posterior wall of the ureter, scarred and fixed to the vessel wall, which was also open. The AUF was occluded by a fresh thrombus. The surgical repair included separation and closure of the right CIA using a monofil running suture, as well as reconstruction of the right ureter with another segment resection and end-to-end anastomosis. A new DJ catheter (4,7 French plastic catheter) was inserted. Postoperatively, the blood count stabilized and the hydronephrosis decreased. The DJ catheter could be removed 3 months later. Eleven months after the major surgery, the patient underwent another semielective laparotomy. The residual tumor had shown slight growth over time and had to be re-resected. The vascular graft in the right CIA was replaced because imaging showed increasing dilatation of the vessel. There was no right-sided hydronephrosis, and the right ureter was intact. Further postoperative follow-up remained unremarkable to date.

## Discussion

AUF is an uncommon and challenging clinical scenario involving the skills and experience of a (pediatric) urologist, a vascular surgeon, and an interventional radiologist. Depending on the severity of bleeding, AUF must be considered a potentially life-threatening condition and may be associated with a high mortality rate. 6 Reviewing the literature, we may notice that the incidence of AUF has risen. Few cases are described in the literature until the turn of the millennium. 6 An increasing number of adult patients with HPS or HPR as well as HVS, the increased application of CIUS, longer postoperative survival, and a generally increased life expectancy may represent contributing factors. 1 4 To the best of our knowledge, we present the first case of AUF in a patient under the age of 18 years. At any age, AUF is still a very rare condition, with a high risk of late diagnosis due to subtle and unspecific symptoms.

AUF can develop with the aorta, CIA, external iliac artery, and hypogastric artery. 6 Concerning etiology, we may differentiate between primary and secondary variants of AUF. 7 Primary AUF is based on vascular diseases such as aneurysms, vascular malformations, etc., and accounts for only a small number of AUFs. In contrast, up to 97.5% of AUFs are secondary and referred to as HPS, HVS, HPR, or CIUS. 1 2 3 7 The awareness and identification of one of these risk factors may help to confirm the diagnosis quickly and may reduce mortality. According to the literature, up to 80% of patients with an AUF had CIUS, 60 to 70% had HPS, and between 50 and 60% had HPR. More than 70% of patients suffered from primary oncological disease, most frequently of gynecological or gastrointestinal origin. 2 3 According to the literature, only adult patients are affected.

The pathophysiology of AUF is not yet fully understood. Maybe localized fibrotic inflammatory reactions due to previous surgery, ureteral splinting, and the presence of an indwelling stent respectively, or radiation at the junction of the vessel and ureter may contribute to the fixation of both structures to each other. The permanent pulsation of the artery against the partially weakened ureteral wall in combination with the fibrotic, ischemic reaction, could lead to local necrosis and consequently to the formation of an AUF. 1 2 3 This might be the possible hypothesis of pathophysiology in our patient.

Making the diagnosis of AUF remains difficult, partly due to non-specific symptoms. Hematuria as the main symptom occurs in many patients and can vary in intensity, from intermittent and hemodynamically irrelevant to massive bleeding, with life-threatening hemorrhagic shock. 1 2 3 4 5 Flank pain, as in our case, or fever may also be observed. However, these symptoms are also very unspecific. 2

Available standard imaging techniques do not detect all cases of AUF. In adult patients, contrast-enhanced computed tomography (CT) angiography represents the preferred method. 6 Concerning pediatric and adolescent patients, no reliable information is available. Angiography may confirm the diagnosis in up to 62%, retrograde pyelography in 51%, and a CT scan in 48% of cases. In terms of sensitivity, there are promising indications for the use of provocative angiography. However, manipulation of the stent or vascular catheter can cause severe bleeding. Consequently, this examination should only be performed in a controlled setting. 3 Negative imaging results do not rule out the presence of an AUF, and the perioperative confirmed diagnosis, as in our case, is no exception. 1 Therefore, AUF must always be excluded in cases of hematuria of unknown origin and previous history of the abovementioned risk factors. 2 In case of doubt, surgical exploration is indicated. Finally, management strategy has to be defined individually based on the specific risk profile of each individual patient. Regardless of the possible serious side effects, vascular endoprosthesis is the best therapeutic option.

Today, there are various techniques available to treat an AUF. In general, we may observe a shift from open surgical repair to minimally invasive endovascular repair. 1 2 3 4 Unsuccessful treatment is usually due to incorrect diagnosis. 1

Since the incidence of AUF is still low, there are no standard operating procedures (SOPs) including a standardized follow-up protocol available. There is no doubt that we have to be aware that the potential long-term outcome may affect pediatric and adolescent patients in a completely different manner compared to adult patients with HPS, HPR, etc. Follow-up after AUF has to be managed on an individual basis. Long-term follow-up of our patient includes outpatient clinic visits to the pediatric urologist and pediatric surgeon, the oncologist, and the vascular surgeon. Depending on the clinical course, follow-up imaging is defined individually based on the specific risk profile of each individual patient.

## Conclusion

In patients with hematuria of unexplained origin and known risk factors in their medical history, AUF has always to be considered and investigated until it has been excluded, as early diagnosis is crucial to treat AUF quickly and reduce mortality. Our case report highlights the rare occurrence of AUF in an adolescent, emphasizing the critical importance of early suspicion and diagnosis, especially in patients with HPS, HVS, HPR, and CIUS. Prompt surgical intervention is essential for preventing life-threatening complications, and clinicians must maintain a high index of suspicion of AUF in cases of unexplained hematuria, even if imaging results are inconclusive.

## References

[1] van den Bergh R CN Moll F L de Vries J PPM Lock T MTW Arterioureteral fistulas: unusual suspects-systematic review of 139 cases Urology 2009 74 02 251 255 19362353 10.1016/j.urology.2008.12.011

[2] Prontera P P Sciorio C De Cillis A Early diagnosis and management of arterio-ureteral ﬁstulas: a literature review Arch Ital Urol Androl 2023 95 01 10928 36924382 10.4081/aiua.2023.10928

[3] Kamphorst K Lock T MTW van den Bergh R CN Arterio-ureteral fistula: systematic review of 445 patients J Urol 2022 207 01 35 43 34555933 10.1097/JU.0000000000002241

[4] Lock T MTW Kamphorst K van den Bergh R CN Arterio-ureteral fistula: a nationwide cross-sectional questionnaire analysis World J Urol 2022 40 03 831 839 35064800 10.1007/s00345-021-03910-3 PMC8783176

[5] Pillai A K Anderson M E Reddick M A Sutphin P D Kalva S P Ureteroarterial fistula: diagnosis and management AJR Am J Roentgenol 2015 204 05 W592-8 25905967 10.2214/AJR.14.13405

[6] Leone L Scarcella S Dell'Atti L Tiroli M Sternardi F Galosi A B Uretero-iliac artery fistula: a challenge diagnosis for a life-threatening condition: monocentric experience and review of the literature Int Urol Nephrol 2019 51 05 789 793 30929222 10.1007/s11255-019-02097-2

[7] Bergqvist D Pärsson H Sherif A Arterio-ureteral fistula–a systematic review Eur J Vasc Endovasc Surg 2001 22 03 191 196 11506509 10.1053/ejvs.2001.1432

